# Eye care human resources: are there gender issues?

**Published:** 2009-06

**Authors:** Paul Courtright

**Affiliations:** Kilimanjaro Centre for Community Ophthalmology PO Box 2254, Moshi, Tanzania.

**Figure:**
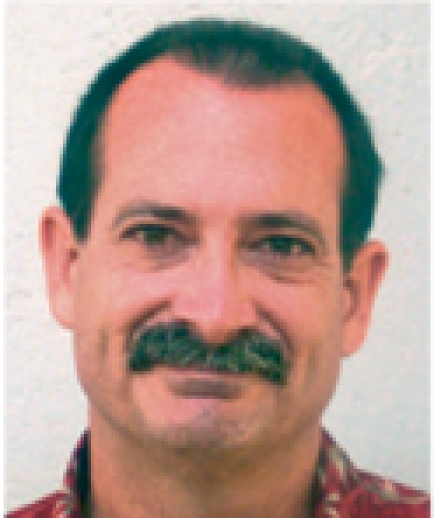


In many settings, women make up a sizeable part of the eye health workforce, whether as distributors of ivermectin for onchocerciasis control, surgeons for trichaisis, ophthalmic nurses and nurse assistants, cataract surgeons, or ophthalmologists. There is a small, but growing, body of literature that suggests that, in the eye health care workforce, men and women may not be supported and paid equally and may not have similar performance levels. Why is this, and what can we do?

## Unequal treatment

In low- and middle-income settings, women's roles include caring for children, producing food, and keeping the family together when their husbands are away from home in order to work or find work. The participation of women in the design and implementation of community eye care programmes is therefore essential.

Despite the intention of the African Programme for Onchocerciasis Control (APOC) to actively encourage participation of women, in most settings the majority of community-selected distributors are men, often because membership in community decision making bodies is male dominated.^1^ It was also noted that female distributors may receive less community support, whether financial or in-kind in nature.^1^ In contrast, the extremely successful Nepal vitamin A programme is built exclusively on female community health volunteers.^2^ Differences may be traced to the fact that vitamin A distribution is targeted at children, traditionally considered to be the responsibility of women, whereas ivermectin benefits men as well as women and children.

## Women's performance and productivity

In eastern Africa, an assessment of productivity of cataract surgeons who were not medical doctors showed that female surgeons were half as productive as male surgeons. Compared to male surgeons, female surgeons were less likely to have adequate nursing support or to have sufficient instruments.^3^ Interviews with female surgeons revealed that they had greater difficulty in negotiating with hospital directors (all men) to obtain the support they needed to do their work. Similarly, in Tanzania, female trichiasis surgeons were less productive (median five operations per year) than their male counterparts (median eight operations per year).^4^

A Fred Hollows Foundation evaluation of their primary eye care programme at health centres in Rwanda suggested that trained female health workers had lower levels of service delivery than their male counterparts. There appeared to be many reasons for this difference, including those related to traditional views of the roles of women and those related to the work environment.

**Figure:**
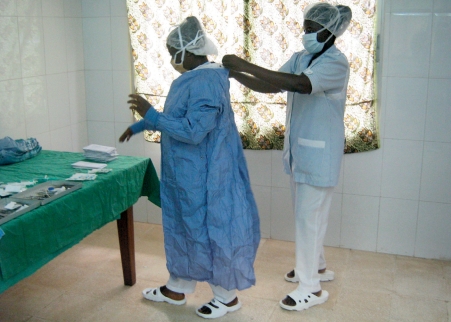
Female eye care workers prepare for surgery. LIBERIA

Joseph Kerkula

Patients' preference to receive health care from one gender or another is commonly reported for female-specific health conditions. May this also be the case for eye related conditions, as reported by Vijayakumar and colleagues in India?^5^

Although often ignored, traditional expectations of women will have a significant impact on how male and female eye care providers are perceived and respected, as well as supported and supervised. We need to understand these norms better and to address any biases we detect, including our own.

In conclusion, it is helpful to note the following:

- Women eye care workers have the same potential to be productive as their male counterparts.
- Community bias against female eye care workers may affect their performance.
- If women eye care workers receive less support than their male counterparts (in terms of access to equipment and nursing support, for example), their performance will suffer.
- Poor performance (resulting from poor support or patient bias) may reinforce negative perceptions of female eye care workers; this may (wrongly) justify both patient bias and the poor support given to them.

By addressing gender issues in human resource development we will strengthen all of our eye care human resources more effectively. Our eye patients will be the ones who benefit.

**What can we do?**

- **Supervisors, managers, and planners** need to be vigilant to ensure equal treatment, support, and access to essential resources for all their eye care staff.
- **Planning of eye care services** needs to take into account any gender biases existing in the community and to make allowances for this; for example, by arranging for female eye care workers to treat female, not male, patients.
- **When promoting eye care services in the community**, it may help to include positive messages about female eye care workers.
